# Supplementary material for: Causal Relationship between Aging and Anorexia Nervosa: A White-Matter-Microstructure-Mediated Mendelian Randomization Analysis
Source: Biomedicines. 2024 Aug 16;12(8):1874. doi: 10.3390/biomedicines12081874 (PMC11351342; doi:10.3390/biomedicines12081874)
Supplement: Supplementary file 1 [file biomedicines-12-01874-s001.zip › Supplementary Figure/Figure S4/Singe SNP plot of MR analysis for TL to ubm-b-1845.pdf]

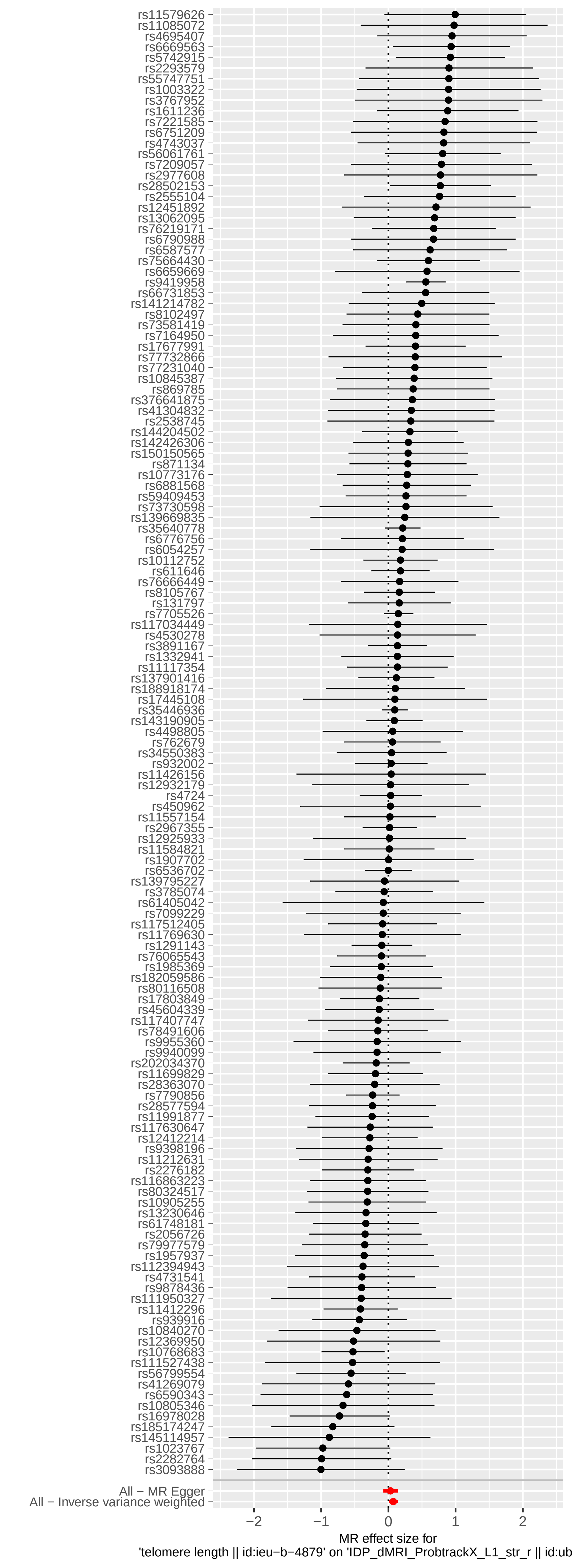
rs11579626
rs11085072
rs4695407
rs6669563
rs5742915
rs2293579
rs55747751
rs1003322
rs3767952
rs1611236
rs7221585
rs6751209
rs4743037
rs56061761
rs7209057
rs2977608
rs28502153
rs2555104
rs12451892
rs13062095
rs76219171
rs6790988
rs6587577
rs75664430
rs6659669
rs9419958
rs66731853
rs141214782
rs8102497
rs73581419
rs7164950
rs17677991
rs77732866
rs77231040
rs10845387
rs869785
rs376641875
rs41304832
rs2538745
rs144204502
rs142426306
rs150150565
rs871134
rs10773176
rs6881568
rs59409453
rs73730598
rs139669835
rs35640778
rs6776756
rs6054257
rs10112752
rs611646
rs76666449
rs8105767
rs131797
rs7705526
rs117034449
rs4530278
rs3891167
rs1332941
rs11117354
rs137901416
rs188918174
rs17445108
rs35446936
rs143190905
rs4498805
rs762679
rs34550383
rs932002
rs11426156
rs12932179
rs4724
rs450962
rs11557154
rs2967355
rs12925933
rs11584821
rs1907702
rs6536702
rs139795227
rs3785074
rs61405042
rs7099229
rs117512405
rs11769630
rs1291143
rs76065543
rs1985369
rs182059586
rs80116508
rs17803849
rs45604339
rs117407747
rs78491606
rs9955360
rs9940099
rs202034370
rs11699829
rs28363070
rs7790856
rs28577594
rs11991877
rs117630647
rs12412214
rs9398196
rs11212631
rs2276182
rs116863223
rs80324517
rs10905255
rs13230646
rs61748181
rs2056726
rs79977579
rs1957937
rs112394943
rs4731541
rs9878436
rs111950327
rs11412296
rs939916
rs10840270
rs12369950
rs10768683
rs111527438
rs56799554
rs41269079
rs6590343
rs10805346
rs16978028
rs185174247
rs145114957
rs1023767
rs2282764
rs3093888
All − MR Egger
All − Inverse variance weighted
−2
−1
0
1
2
MR effect size for
'telomere length || id:ieu−b−4879' on 'IDP_dMRI_ProbtrackX_L1_str_r || id:ub
